# Genetic association of *ANGPT2* with primary open-angle glaucoma

**DOI:** 10.1186/s40662-022-00309-y

**Published:** 2022-10-06

**Authors:** Jing Na He, Tsz Kin Ng, Shi Yao Lu, Pancy Oi Sin Tam, Poemen P. Chan, Clement C. Tham, Chi Pui Pang, Li Jia Chen, Wai Kit Chu

**Affiliations:** 1 Department of Ophthalmology & Visual Sciences, The Chinese University of Hong Kong, Hong Kong, China; 2 Joint Shantou International Eye Center of Shantou University and The Chinese University of Hong Kong, Shantou, Guangdong China; 3 Shantou University Medical College, Shantou, Guangdong China; 4 Lam Kin Chung, Jet King-Shing Ho Glaucoma Treatment and Research Centre, The Chinese University of Hong Kong, Hong Kong, China; 5 Lim Por-Yen Eye Genetics Research Centre, The Chinese University of Hong Kong, Hong Kong, China; 6 Department of Ophthalmology & Visual Sciences, The Chinese University of Hong Kong, Hong Kong Eye Hospital, 147K Argyle Street, Kowloon, Hong Kong China

**Keywords:** Primary open-angle glaucoma, Normal tension glaucoma, ANGPT2

## Abstract

**Background:**

To determine the association of the *ANGPT2* gene with primary open-angle glaucoma (POAG) in Chinese.

**Methods:**

Six single-nucleotide polymorphisms (SNPs) in *ANGPT2* (rs2515487, rs2922869, rs13255574, rs4455855, rs13269021, and rs11775442) were genotyped in a total of 2601 study subjects from two cohorts. One is a Hong Kong Chinese cohort of 484 high tension glaucoma (HTG) and 537 normal tension glaucoma (NTG) patients, and 496 non-glaucoma control subjects. Another cohort is a Shantou Chinese cohort of 403 HTG and 135 NTG patients, and 543 non-glaucoma control subjects. Subgroup analysis by sex was conducted. Outcomes from different cohorts were combined for meta-analysis.

**Results:**

The association of SNP rs11775442 with NTG in the Hong Kong cohort [*P* = 0.0498, OR = 1.24, 95% confidence interval (CI) 1.00–1.55] after adjusting for age and sex did not reach statistical significance after Bonferroni correction. Other SNPs were not significantly associated with NTG, HTG and POAG in individual cohort or in the combined analyses (*P* > 0.05). In the subgroup analysis by sex, SNP rs13269021 in the Shantou cohort, but not in the Hong Kong cohort, was significantly associated with NTG in males (*P* = 0.0081, OR = 1.67, 95% CI: 1.14–2.43) but not in females (*P* = 0.874). In the combined analyses by sex, no SNPs were significantly associated with NTG, HTG and POAG.

**Conclusions:**

In the subgroup analysis by sex, a significant association was shown in SNP rs13269021 with NTG in Shantou males, but not in Hong Kong males. Further studies are needed to verify the association between *ANGPT2* locus (rs13269021) and NTG in Chinese males.

## Background

Glaucoma is a leading cause of irreversible blindness worldwide [1]. In 2010, glaucoma caused 2.1 million (6.5%) out of a total 32.4 million blindness globally [2]. Primary open-angle glaucoma (POAG) is the most common type of glaucoma. According to the highest detected intraocular pressure (IOP), POAG can be classified into high tension POAG (IOP > 21 mmHg) and normal tension POAG (IOP ≤ 21 mmHg).

POAG is a multifactorial progressive optic neuropathy. Both genetic and environmental factors are related to the etiology of POAG. Until now, four genes have been reported to be causative or probably causative of POAG: myocilin (*MYOC*, also called *GLC1A*) [3, 4], optineurin (*OPTN*, also called *GLC1E*) [5, 6], neurotrophin 4 (*NTF4*) [7, 8] and tank-binding kinase 1 (*TBK1*, also called *GLC1P*) [9–11].

IOP is a major risk factor for POAG and lowering IOP is currently the only intervention that has been proven to be able to delay the progression of glaucoma in human [12, 13]. Trabecular meshwork/Schlemm’s canal (TM/SC) conventional aqueous humor (AH) outflow pathway plays a key role in the regulation of IOP [14]. Previous studies found that the SC tissues show features of lymphatic vessels including expressing lymphatic regulator PROX1, lymphangiogenic growth factor VEGF-C and its receptor VEGFR-3, suggesting lymphangiogenesis induction could be developed as a novel treatment of glaucoma [15–18]. Tie receptors and their angiopoietin (Angpt) ligands are key regulators of vascular morphogenesis and homeostasis [19]. There are six members in the Tie-Angpt system: TIE1, TIE2, ANGPT1, ANGPT2, ANGPT3 and ANGPT4. ANGPT2 acts as an agonist and an antagonist to TIE2 in a context-dependent manner [20]. Deletion of Angpt1/Angpt2 or Tie2 in adult mice severely impaired SC integrity and transcytosis, leading to hallmarks of POAG including elevated IOP, retinal neuron damages, and impairment of retinal ganglion cell functions [21]. A POAG genome-wide meta-analysis in 2021 identified the association between an intronic variant rs2515437 in *ANGPT2* and POAG [22]. Another *ANGPT2* SNP, rs76020419, has been reported to be associated with IOP [23, 24]. Therefore, we hypothesize that *ANGPT2* is a potential gene associated with POAG. However, both rs2515437 and rs76020419 are monomorphic in East Asians. Therefore, we aim to study other POAG-associated variants in this gene in East Asians, especially in our local population. Previously, we reported six *ANGPT2* SNPs that were associated with neovascular age-related macular degeneration (nAMD) and polypoidal choroidal vasculopathy (PCV) [25]. As nAMD and PCV are related to retinal vessel abnormalities, we hypothesize these six *ANGPT2* SNPs may also associate with POAG, in which SC is also a lymphatic-like vessel. In this study, we performed a SNP association analysis to determine the association of these six *ANGPT2* SNPs with POAG.

## Methods

### Study participants

This study involved 2601 unrelated participants from two independent Chinese cohorts. One cohort is a Hong Kong Chinese cohort of 484 high tension glaucoma (HTG) and 537 normal tension glaucoma (NTG) patients, and 496 non-glaucoma control subjects. Another cohort is a Shantou Chinese cohort of 403 HTG and 135 NTG patients, and 543 non-glaucoma control subjects. All participants are Han Chinese, recruited from the eye clinics of the Prince of Wales Hospital, the Hong Kong Eye Hospital and the Joint Shantou International Eye Center. The study protocol was approved by the institutional ethics committee at the respective institutions (KC/KE-18-0045/ER-3). Written informed consent was obtained from each participant. The study procedures were performed in accordance with the tenets of the Declaration of Helsinki.

Diagnosis was based on meeting all the following criteria: exclusion of secondary causes of glaucoma (steroid-induced glaucoma, neovascular glaucoma, uveitis or trauma); anterior chamber angle open (grade III or IV on gonioscopy); characteristic optic disc changes (vertical cup-to-disc ratio > 0.5, disc hemorrhage, or thin/notched neuroretinal rim); and characteristic visual field changes with reference to the Anderson’s criteria for minimal abnormality in glaucoma [26]. IOP was measured by using applanation tonometry and visual field was evaluated by a perimeter (Humphrey Field Analyzer; Carl Zeiss Meditec, Dublin, CA) using the Glaucoma Hemifield Test. Patients whose highest recorded IOP lower than 21 mmHg without medication were regarded as NTG patients. Patients whose highest recorded IOP higher than or equal to 21 mmHg without medication were regarded as HTG patients. Patients with congenital glaucoma were excluded. People who attended the clinic for conditions other than glaucoma, including mild senile cataract, floaters, mild refractive errors and itchy eyes were recruited as non-glaucoma control subjects.

### Single-nucleotide polymorphisms (SNPs) selection and genotyping

Six SNPs in *ANGPT2* (rs2515487, rs2922869, rs13255574, rs4455855, rs13269021, and rs11775442) were reported as suggestive disease-association in nAMD and PCV [25]. These six SNPs are independent of each other. They were selected for genotyping assays in POAG patients from the Hong Kong and Shantou cohorts. Genomic DNA from peripheral blood was extracted using a QIAamp Blood Kit (Qiagen, Hilden, Germany) according to the manufacturer’s protocol. SNPs were genotyped using TaqMan genotyping assays (Applied Biosystems, Foster City, CA, USA) with a Roche LightCycle 480 Real-Time PCR System (Roche Diagnostics; Basel, Switzerland) following the manufacturer’s instructions.

### Statistical analysis

Hardy-Weinberg equilibrium (HWE) of each SNP in the non-glaucoma controls was assessed using the χ^2^ test in PLINK (v.1.07; available in the public domain at http://pngu.mgh.harvard.edu/purcell/plink/). Allelic and genotypic distributions were compared by the χ^2^ test between cases and controls among different study cohorts. The odds ratio (OR) and 95% confidence intervals (CI) for each SNP were calculated. Logistic regression analysis was used to evaluate the genetic effects of the SNPs adjusted for age and sex.

To combine the data from the two study cohorts, we adopted the Mantel-Haenszel χ^2^ test to obtain the combined ORs and 95% CIs for all SNPs. The test was performed using Review Manager (RevMan, version 5.2; The Cochrane Collaboration, Copenhagen, Denmark).

In this study, we adopted the Bonferroni method to correct the *P* values in multiple testing. A final *P* value of less than 0.0083 (0.05/6, where 6 was the number of SNPs included in data analysis) would be required to conclude a significant disease association.

## Results

### Demographics and quality control outcomes

Table 1 shows the demographics of the POAG patients and non-glaucoma controls. Mean ages and sex ratios were statistically different between POAG cases and non-glaucoma control group in the two cohorts (*P* < 0.05; Table 1). Therefore, age and sex were adjusted in logistic regressions. In the Shantou cohort, all SNPs conformed to HWE (*P* > 0.05) in the non-glaucoma control group with call rates ≥ 98%. In the Hong Kong group, the rs2922869 showed deviation from HWE (*P* < 0.05) and thus was excluded from further analysis.

**Table 1:** Demographics of study subjects

	POAG	HTG	NTG	Non-glaucoma control	^a^*P*
Hong Kong					
N	1021	484	537	496	
Age (years)	62.0 ± 14.0	62.0 ± 15.3	63.2 ± 12.8	70.2 ± 10.8	< 0.05
Sex (male %)	56.10%	61.00%	51.80%	37.10%	< 0.05
Shantou					
N	541	403	135	543	
Age (years)	55.9 ± 18.6	54.0 ± 19.4	61.6 ± 14.6	74.4 ± 6.9	< 0.05
Sex (male %)	68.40%	71.70%	58.50%	52.10%	< 0.05

*HTG* = high tension glaucoma; *NTG* = normal tension glaucoma; *POAG* = primary open-angle glaucoma

^a^*P* value: mean age between cases and controls was compared using independent t*-*test; sex proportion between cases and controls was compared with the *χ*^*2*^ test

### Association of *ANGPT2* with POAG

There was no significant association of all the six SNPs with NTG, HTG and POAG in individual cohorts or in the combined analyses after Bonferroni correction (*P* > 0.0083, Table 2). Only one SNP, rs11775442, showed a borderline association with NTG in the Hong Kong cohort at the level of *P* value < 0.05 (*P* = 0.0498, OR = 1.24, 95% CI 1.00–1.55) after adjusting for age and sex (Table 2).

**Table 2:** Association of SNPs from *ANGPT2* in NTG, HTG and POAG adjusted for age and sex

SNP	Nucleotide change	Risk allele	RAF				HTG vs. Control				NTG vs. Control				POAG vs. Control			
			HTG (n = 484)	NTG (n = 537)	POAG (n = 1021)	Control (n = 496)	*P*_non-adjusted_	Non-adjusted OR (95% CI)	*P*_adjusted_	Adjusted OR (95% CI)	*P*_non-adjusted_	Non-adjusted OR (95% CI)	*P*_adjusted_	Adjusted OR (95% CI)	*P*_non-adjusted_	Non-adjusted OR (95% CI)	*P*_adjusted_	Adjusted OR (95% CI)
Hong Kong																		
rs2515487	c.289-10440C>A	A	0.240	0.238	0.239	0.239	0.950	1.01 (0.82–1.24)	0.815	1.03 (0.82–1.28)	0.977	1.00 (0.81–1.22)	0.761	1.03 (0.84–1.28)	0.986	1.00 (0.84–1.20)	0.704	1.04 (0.86–1.25)
rs13255574	c.289-8669C>T	C	0.789	0.789	0.789	0.762	0.148	1.17 (0.95–1.45)	0.367	1.11 (0.89–1.38)	0.148	1.16 (0.95–1.43)	0.118	1.19 (0.96–1.47)	0.093	1.17 (0.97–1.40)	0.173	1.14 (0.95–1.37)
rs4455855	c.289-7422T>A	A	0.300	0.29	0.295	0.293	0.740	1.03 (0.85–1.26)	0.833	1.02 (0.83–1.26)	0.850	0.98 (0.81–1.19)	0.739	1.03 (0.85–1.26)	0.943	1.01 (0.85–1.19)	0.882	1.01 (0.85–1.21)
rs13269021	c.289-6755G>T	G	0.543	0.54	0.541	0.523	0.389	1.08 (0.90–1.29)	0.715	1.04 (0.86–1.25)	0.441	1.07 (0.90–1.27)	0.446	1.07 (0.90–1.29)	0.348	1.08 (0.92–1.25)	0.542	1.05 (0.90–1.23)
rs11775442	c.289-6350A>G	A	0.782	0.796	0.789	0.764	0.354	1.11 (0.89–1.37)	0.558	1.07 (0.86–1.33)	0.083	1.20 (0.98–1.48)	0.0498*	1.24 (1.00–1.55)	0.119	1.16 (0.96–1.38)	0.149	1.15 (0.95–1.39)
Shantou																		
rs2515487	c.289-10440C>A	A	0.286	0.282	0.285	0.286	0.981	1.00 (0.82–1.23)	0.995	1.00 (0.76–1.31)	0.908	0.98 (0.73–1.32)	0.971	1.01 (0.71–1.43)	0.989	1.00 (0.83–1.21)	0.916	0.99 (0.78–1.26)
rs2922869	c.289-9785T>C	T	0.729	0.727	0.727	0.704	0.251	1.13 (0.92–1.39)	0.439	1.12 (0.85–1.47)	0.458	1.12 (0.83–1.52)	0.529	1.12 (0.78–1.60)	0.247	1.12 (0.92–1.36)	0.407	1.11 (0.87–1.42)
rs13255574	c.289-8669C>T	C	0.785	0.782	0.782	0.763	0.276	1.13 (0.91–1.42)	0.320	1.16 (0.86–1.57)	0.530	1.11 (0.80–1.54)	0.607	1.11 (0.75–1.63)	0.297	1.12 (0.91–1.37)	0.380	1.13 (0.86–1.46)
rs4455855	c.289-7422T>A	A	0.323	0.308	0.319	0.330	0.735	0.97 (0.79–1.18)	0.776	0.96 (0.74–1.25)	0.503	0.91 (0.68–1.21)	0.633	0.92 (0.66–1.29)	0.576	0.95 (0.79–1.14)	0.558	0.93 (0.74–1.18)
rs13269021	c.289-6755G>T	G	0.584	0.626	0.594	0.500	0.354	1.09 (0.91–1.32)	0.217	1.17 (0.91–1.49)	0.059	1.31 (0.99–1.73)	0.183	1.25 (0.90–1.72)	0.137	1.14 (0.96–1.36)	0.142	1.18 (0.95–1.47)
rs11775442	c.289-6350A>G	A	0.808	0.788	0.802	0.796	0.535	1.07 (0.86–1.35)	0.357	1.15 (0.85–1.55)	0.803	0.96 (0.70–1.32)	0.915	1.02 (0.70–1.49)	0.734	1.04 (0.84–1.28)	0.586	1.08 (0.83–1.40)

*CI* = confidence interval; *HTG* = high tension glaucoma; *NTG* = normal tension glaucoma; *OR* = odds ratio; *POAG* = primary open-angle glaucoma; *RAF* = risk allele frequency

*P*_non-adjusted_ = *P* values derived from the χ^2^ test

*P*_adjusted_ = *P* values derived from logistic regression models after adjusting for age and sex

^*^P value < 0.05

**Fig. 1:**
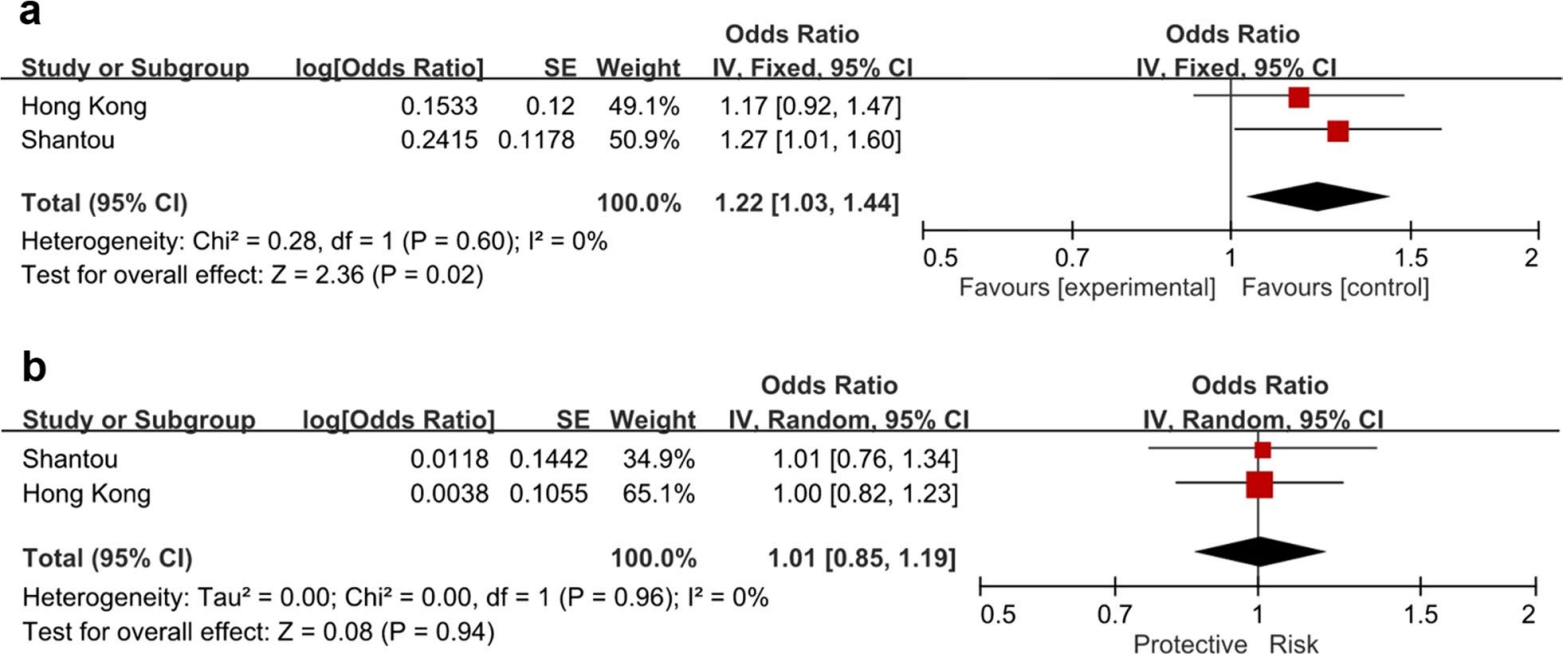
Association of rs1326901 with POAG using meta‐analysis in males (**a**) and females (**b**) combining the Hong Kong and Shantou cohorts. The association of rs13269021 in POAG was non-significant after combination by meta-analysis (*P* = 0.020 in males and 0.94 in females). CI, confidence interval; IV, inverse variance

In the subgroup analysis by sex, the top association was shown in SNP rs13269021 with NTG in Shantou males (*P* = 0.0081, OR = 1.67, 95% CI: 1.14–2.43; Table 3) but not in females (*P* = 0.874). With regards to the glaucoma subtypes, *P* values of this SNP were 0.040 in POAG (OR = 1.27) and 0.197 in HTG (OR = 1.17), which could not withstand the Bonferroni correction. Other associations were also non-significant after Bonferroni correction (*P* > 0.0083). Some of the SNP associations showed differences between male and female subgroups. In the Hong Kong cohort, the associations of rs13255574 in HTG (*P* = 0.010) and POAG (*P* = 0.017) in males could not withstand the Bonferroni correction, while *P* values of the associations were high in females i.e., HTG and POAG (*P* = 0.605 and 0.824, respectively). In the Shantou cohort, no SNP was significantly associated with NTG, HTG and POAG in both sexes. The meta-analysis of Hong Kong and Shantou subjects showed a slight difference between the sexes. The association of rs13269021 in POAG was still non-significant in meta-analysis (*P* = 0.020 in males and 0.94 in females, Fig. 1). In the combined analyses by sex, no SNP was significantly associated with NTG, HTG and POAG (*P* > 0.05).

**Table 3:** Association of SNPs from *ANGPT2* in females and males with NTG, HTG and POAG

SNP	Nucleotide change	Risk allele	RAF								HTG vs. Control				NTG vs. Control				POAG vs. Control			
			HTG	HTG	NTG	NTG	POAG	POAG	Control	Control	*P*_male_	Male OR (95% CI)	*P*_female_	Female OR (95% CI)	*P*_male_	OR_male_ (95% CI)	*P*_female_	OR_female_ (95% CI)	*P*_male_	OR_male_ (95% CI)	*P*_female_	OR_female_ (95% CI)
			Male	Female	Male	Female	Male	Female	Male	Female												
			(n = 295)	(n = 189)	(n = 278)	(n = 259)	(n = 573)	(n = 448)	(n = 184)	(n = 312)												
Hong Kong																						
rs2515487	c.289-10440C>A	A	0.238	0.243	0.212	0.266	0.226	0.257	0.223	0.248	0.586	1.09 (0.80–1.49)	0.857	0.97 (0.72–1.31)	0.702	0.94 (0.68–1.29)	0.488	1.10 (0.84–1.43)	0.914	1.02 (0.77–1.35)	0.714	1.05 (0.83–1.32)
rs13255574	c.289-8669C>T	C	0.805	0.765	0.781	0.797	0.793	0.784	0.734	0.779	0.010*	1.50 (1.10–2.04)	0.605	0.92 (0.68–1.25)	0.101	1.29 (0.95–1.75)	0.448	1.12 (0.84–1.49)	0.017*	1.39 (1.06–1.83)	0.824	1.03 (0.80–1.32)
rs4455855	c.289-7422T>A	A	0.307	0.290	0.29	0.290	0.298	0.29	0.272	0.306	0.247	1.19 (0.89–1.58)	0.588	0.93 (0.70–1.23)	0.556	1.09 (0.81–1.47)	0.544	0.92 (0.72–1.19)	0.327	1.14 (0.88–1.48)	0.492	0.92 (0.74–1.16)
rs13269021	c.289-6755G>T	G	0.553	0.527	0.545	0.535	0.549	0.531	0.511	0.530	0.207	1.18 (0.91–1.54)	0.910	0.99 (0.76–1.27)	0.307	1.15 (0.88–1.49)	0.885	1.02 (0.81–1.29)	0.201	1.17 (0.92–1.47)	0.971	1.00 (0.82–1.23)
rs11775442	c.289-6350A>G	A	0.781	0.782	0.784	0.808	0.783	0.797	0.767	0.766	0.461	1.12 (0.82–1.53)	0.555	1.10 (0.81–1.49)	0.406	1.14 (0.83–1.56)	0.085	1.29 (0.97–1.71)	0.381	1.13 (0.86–1.49)	0.146	1.20 (0.94–1.54)
Shantou																						
rs2515487	c.289-10440C>A	A	0.295	0.263	0.295	0.264	0.296	0.262	0.298	0.272	0.909	0.99 (0.76–1.27)	0.808	0.96 (0.66–1.38)	0.936	0.98 (0.67–1.45)	0.864	0.96 (0.61–1.52)	0.937	0.99 (0.78–1.26)	0.746	0.95 (0.69–1.30)
rs2922869	c.289-9785T>C	T	0.712	0.770	0.721	0.736	0.713	0.757	0.713	0.695	0.980	1.00 (0.77–1.30)	0.037	1.49 (1.02–2.16)	0.849	1.04 (0.70–1.55)	0.383	1.23 (0.77–1.98)	0.999	1.00 (0.78–1.29)	0.051	1.37 (1.00–1.88)
rs13255574	c.289-8669C>T	C	0.771	0.821	0.799	0.759	0.775	0.798	0.765	0.760	0.809	1.04 (0.78–1.37)	0.082	1.44 (0.95–2.16)	0.394	1.22 (0.77–1.91)	0.98	0.99 (0.62–1.59)	0.668	1.06 (0.81–1.39)	0.226	1.23 (0.88–1.72)
rs4455855	c.289-7422T>A	A	0.339	0.282	0.346	0.255	0.341	0.271	0.340	0.319	0.960	0.99 (0.78–1.27)	0.300	0.83 (0.58–1.18)	0.889	1.03 (0.71–1.49)	0.193	0.74 (0.46–1.17)	0.981	1.00 (0.79–1.27)	0.128	0.78 (0.57–1.07)
rs13269021	c.289-6755G>T	G	0.577	0.602	0.658	0.580	0.596	0.591	0.539	0.443	0.197	1.17 (0.92–1.49)	0.733	1.06 (0.77–1.46)	0.008*	1.67 (1.14–2.43)	0.874	0.97 (0.63–1.48)	0.040	1.27 (1.01–1.60)	0.934	1.01 (0.76–1.34)
rs11775442	c.289-6350A>G	A	0.798	0.833	0.796	0.778	0.796	0.813	0.793	0.798	0.870	1.02 (0.77–1.36)	0.272	1.26 (0.84–1.89)	0.946	1.02 (0.66–1.57)	0.652	0.90 (0.55–1.45)	0.900	1.02 (0.78–1.33)	0.597	1.10 (0.78–1.54)

*CI* = confidence interval; *HTG* = high tension glaucoma; *NTG* = normal tension glaucoma; *OR* = odds ratio; *POAG* = primary open-angle glaucoma; *RAF* = risk allele frequency

^*^P value < 0.05

## Discussion

In this study, all six SNPs in *ANGPT2* were not significantly associated with NTG, HTG and POAG in individual cohorts or in the combined analyses. Only SNP rs11775442 showed a nominal association with NTG in the Hong Kong cohort (*P* = 0.0498, OR = 1.24, 95% CI: 1.00–1.55) after adjusting for age and sex. It has been reported that rs11775442 (nAMD: *P* = 0.016; OR = 1.43; 95% CI: 1.0–1.92; PCV: *P* = 0.044; OR = 1.34; 95% CI: 1.01–1.79) were nominally associated with nAMD and PCV in the Hong Kong cohort [25]. Currently, no significant association has been reported between rs11775442 and any other disease. Further studies are needed to verify the association between SNP 11775442 with NTG.

In the subgroup analysis by sex, SNP rs13269021 in the Shantou cohort was significantly associated with NTG in males but not in females. Therefore, further studies to confirm its sex‐specific effects are warranted. Inconsistent results have been reported on the association of sex in POAG. In the United States, no significant sex difference in the POAG prevalence could be detected [27–29]. In contrast, a study from Netherlands reported a three-times higher POAG risk in males, while POAG was found more prevalent in females in an Australian study [30, 31]. In another study in African Americans, males showed a higher prevalence of POAG than females [32]. In the Los Angeles Latino Eye Study, males were found to possess an OR of 1.73 of having glaucoma vs. females [33]. Mechanistically, female sex hormones have been suggested to be protective against POAG [34, 35]. Serum estradiol levels were found positively correlated with the blood flow in the ophthalmic artery [36]. Menopause women receiving estradiol 17-valerate hormone replacement therapy showed significantly lower blood flow resistance in their central retinal arteries [37]. In addition, 17β-estradiol was also shown to protect retinal ganglion cells in rat and mouse glaucoma models [38, 39]. Interestingly, 17β-estradiol and another female sex hormone medroxyprogesterone acetate were able to reduce the protein production of ANGPT2 in human endometrial stromal cells [40]. Apart from our current study reporting *ANGPT2* SNP (rs13269021) associating with male NTG in the Shantou cohort, a previous study found that BDNF Val66Met was associated with slower progression of POAG in females [41]. To our knowledge, our results are the first to highlight a sex-specific association of *ANGPT2* in POAG.

The angiopoietin-Tie2 (Angpt-Tie2) pathway has been reported to control vascular maturation and stabilization [42]. Despite much research over the past decade, the roles of Angpt2 in the Angpt-Tie2 signaling system and vascular biology are still not fully understood. It is unclear whether Angpt2 is an antagonist or agonist of Tie2 in terms of vascular remodeling [42]. Better understanding of Angpt2 is very important to translate the use of its specific inhibitors alone or in combination with other anti-angiogenic agents into potential clinical treatments.

Regarding the SC, it has been reported that disruption of the Angpt-Tie2 signaling pathway results in high IOP, buphthalmos, and classical features of glaucoma, including retinal ganglion cell degeneration and vision loss in mice [21, 43]. Impairment of this system could be involved in the adult-onset POAG, as well as in the primary congenital glaucoma [44]. In our study, all six SNPs in *ANGPT2* have not been found to be significantly associated with NTG, HTG and POAG in individual cohorts or in the combined analyses. Although Angpt-Tie2 signaling pathway is essential in the SC outflow pathway, Angpt2 was reported to play an indirect role as an agonist and an antagonist to Tie2 depending on the expression of an endothelial-specific phosphatase VEPTP [20]. Therefore, the genetic association between Angpt2 and POAG could also be influenced by the expression of other genes. For example, as a binding partner of Angpt2, a *SVEP1* missense allele (rs61751937) has been reported to associate with POAG [22, 45]. According to the HapMap Genome Browser release #27 dataset, 34 SNPs could be selected in the locus of *ANGPT2* [25]. In this study six SNPs were selected as they were reported as suggestive disease-associations in nAMD and PCV [25]. All these six SNPs are located in the introns of *ANGPT2* [22]. Interestingly, instead of these six SNPs, SNP rs76020419 and rs2515437 have been reported to associate with POAG and elevated IOP [22–24]. rs76020419 is located in the 3ʹ untranslated region of *ANGPT2*, as well as in an intron of *MCPH1*. rs2515437 is located in the intron of *ANGPT2* and *MCPH1*. *MCPH1* encodes a microcephalin protein that is important for brain development [46]. Haplotype‐tagging SNP analysis of *ANGPT2* and *MCPH1* should be further studied in POAG patients.

In the subgroup analysis, our results indicated a male-specific association in rs13269021 in NTG from the Shantou cohort. In this cohort, rs13269021 conferred approximately 1.67-fold increased risk of NTG in males. But the OR of the SNP in females was 0.97, indicating a slightly decreased risk of NTG. The same effect has not been found in the Hong Kong cohort although the sample size of NTG patients from the Hong Kong cohort was larger than the Shantou cohort. Therefore, the sex-specific effects of this locus remained to be determined in more NTG samples.

## Limitations

There are several limitations in this study. First, these six SNPs chosen according to our previous nAMD and PCV study could not cover the genetic linkage disequilibrium regions in the *ANGPT2* gene. It may have narrowed the scope of the findings. Second, the key finding of rs13269021 in the Shantou cohort significantly associated with NTG in males but not in females could not be reproduced in the Hong Kong cohort. Our cohorts were limited to ethnic Chinese. Therefore, our results may not be translatable to other ethnicities. Third, all six SNPs are located in introns of *ANGPT2*. The functions of these SNPs are unclear. Indeed, no publication have reported that these six SNPs could cause any observable phenotypes.

## Conclusions

In the subgroup analysis by sex, a significant association was shown in SNP rs13269021 with NTG in Shantou males, but not in Hong Kong males. Further studies are needed to verify the association between *ANGPT2* locus (rs13269021) and NTG in Chinese males.

## Data Availability

The datasets supporting the conclusions of this article are included within the article.

## References

[1] Pascolini D, Mariotti SP (2012). Global estimates of visual impairment: 2010. Br J Ophthalmol.

[2] Bourne RR, Taylor HR, Flaxman SR, Keeffe J, Leasher J, Naidoo K (2016). Number of people blind or visually impaired by glaucoma worldwide and in world regions 1990–2010: a meta-analysis. PLoS One.

[3] Sheffield VC, Stone EM, Alward WL, Drack AV, Johnson AT, Streb LM (1993). Genetic linkage of familial open angle glaucoma to chromosome 1q21–q31. Nat Genet.

[4] Stone EM, Fingert JH, Alward WL, Nguyen TD, Polansky JR, Sunden SL (1997). Identification of a gene that causes primary open angle glaucoma. Science.

[5] Rezaie T, Child A, Hitchings R, Brice G, Miller L, Coca-Prados M (2002). Adult-onset primary open-angle glaucoma caused by mutations in optineurin. Science.

[6] Sarfarazi M, Child A, Stoilova D, Brice G, Desai T, Trifan OC (1998). Localization of the fourth locus (GLC1E) for adult-onset primary open-angle glaucoma to the 10p15-p14 region. Am J Hum Genet.

[7] Pasutto F, Matsumoto T, Mardin CY, Sticht H, Brandstätter JH, Michels-Rautenstrauss K (2009). Heterozygous NTF4 mutations impairing neurotrophin-4 signaling in patients with primary open-angle glaucoma. Am J Hum Genet.

[8] Vithana EN, Nongpiur ME, Venkataraman D, Chan SH, Mavinahalli J, Aung T (2010). Identification of a novel mutation in the NTF4 gene that causes primary open-angle glaucoma in a Chinese population. Mol Vis.

[9] Bennett SR, Alward WL, Folberg R (1989). An autosomal dominant form of low-tension glaucoma. Am J Ophthalmol.

[10] Fingert JH, Robin AL, Stone JL, Roos BR, Davis LK, Scheetz TE (2011). Copy number variations on chromosome 12q14 in patients with normal tension glaucoma. Hum Mol Genet.

[11] Ritch R, Darbro B, Menon G, Khanna CL, Solivan-Timpe F, Roos BR (2014). TBK1 gene duplication and normal-tension glaucoma. JAMA Ophthalmol.

[12] Jonas JB, Aung T, Bourne RR, Bron AM, Ritch R, Panda-Jonas S (2017). Glaucoma. Lancet.

[13] Leske MC, Heijl A, Hussein M, Bengtsson B, Hyman L, Komaroff E (2003). Factors for glaucoma progression and the effect of treatment: the early manifest glaucoma trial. Arch Ophthalmol.

[14] Llobet A, Gasull X, Gual A (2003). Understanding trabecular meshwork physiology: a key to the control of intraocular pressure?. News Physiol Sci.

[15] Truong TN, Li H, Hong YK, Chen L (2014). Novel characterization and live imaging of Schlemm’s canal expressing Prox-1. PLoS One.

[16] Aspelund A, Tammela T, Antila S, Nurmi H, Leppanen VM, Zarkada G (2014). The Schlemm's canal is a VEGF-C/VEGFR-3-responsive lymphatic-like vessel. J Clin Invest.

[17] Kizhatil K, Ryan M, Marchant JK, Henrich S, John SW (2014). Schlemm's canal is a unique vessel with a combination of blood vascular and lymphatic phenotypes that forms by a novel developmental process. PLoS Biol.

[18] Park DY, Lee J, Park I, Choi D, Lee S, Song S (2014). Lymphatic regulator PROX1 determines Schlemm's canal integrity and identity. J Clin Invest.

[19] Augustin HG, Koh GY, Thurston G, Alitalo K (2009). Control of vascular morphogenesis and homeostasis through the angiopoietin-Tie system. Nat Rev Mol Cell Biol.

[20] Souma T, Thomson BR, Heinen S, Carota IA, Yamaguchi S, Onay T (2018). Context-dependent functions of angiopoietin 2 are determined by the endothelial phosphatase VEPTP. Proc Natl Acad Sci USA.

[21] Kim J, Park DY, Bae H, Park DY, Kim D, Lee CK (2017). Impaired angiopoietin/Tie2 signaling compromises Schlemm's canal integrity and induces glaucoma. J Clin Invest.

[22] Gharahkhani P, Jorgenson E, Hysi P, Khawaja AP, Pendergrass S, Han X (2021). Genome-wide meta-analysis identifies 127 open-angle glaucoma loci with consistent effect across ancestries. Nat Commun.

[23] Khawaja AP, Cooke Bailey JN, Wareham NJ, Scott RA, Simcoe M, Igo RP (2018). Genome-wide analyses identify 68 new loci associated with intraocular pressure and improve risk prediction for primary open-angle glaucoma. Nat Genet.

[24] MacGregor S, Ong JS, An J, Han X, Zhou T, Siggs OM (2018). Genome-wide association study of intraocular pressure uncovers new pathways to glaucoma. Nat Genet.

[25] Ma L, Brelen ME, Tsujikawa M, Chen H, Chu WK, Lai TY (2017). Identification of ANGPT2 as a new gene for neovascular age-related macular degeneration and polypoidal choroidal vasculopathy in the Chinese and Japanese populations. Invest Ophthalmol Vis Sci.

[26] Anderson DR, Patella VM (1999). Automated static perimetry.

[27] Friedman DS, Wolfs RC, O'Colmain BJ, Klein BE, Taylor HR, West S (2004). Prevalence of open-angle glaucoma among adults in the United States. Arch Ophthalmol.

[28] Tielsch JM, Sommer A, Katz J, Royall RM, Quigley HA, Javitt J (1991). Racial variations in the prevalence of primary open-angle glaucoma. The Baltimore Eye Survey. JAMA.

[29] Klein BE, Klein R, Sponsel WE, Franke T, Cantor LB, Martone J (1992). Prevalence of glaucoma. The Beaver Dam Eye Study. Ophthalmology.

[30] Dielemans I, Vingerling JR, Wolfs RC, Hofman A, Grobbee DE, de Jong PT (1994). The prevalence of primary open-angle glaucoma in a population-based study in the Netherlands. The Rotterdam Study. Ophthalmology.

[31] Mitchell P, Smith W, Attebo K, Healey PR (1996). Prevalence of open-angle glaucoma in Australia. The Blue Mountains Eye Study. Ophthalmology.

[32] Leske MC, Connell AM, Schachat AP, Hyman L (1994). The Barbados Eye Study. Prevalence of open angle glaucoma. Arch Ophthalmol.

[33] Doshi V, Ying-Lai M, Azen SP, Varma R, Los Angeles Latino Eye Study Group (2008). Sociodemographic, family history, and lifestyle risk factors for open-angle glaucoma and ocular hypertension. The Los Angeles Latino Eye Study. Ophthalmology.

[34] Lee AJ, Mitchell P, Rochtchina E, Healey PR, Blue Mountains Eye Study (2003). Female reproductive factors and open angle glaucoma: the Blue Mountains Eye Study. Br J Ophthalmol.

[35] Pasquale LR, Rosner BA, Hankinson SE, Kang JH (2007). Attributes of female reproductive aging and their relation to primary open-angle glaucoma: a prospective study. J Glaucoma.

[36] Toker E, Yenice O, Akpinar I, Aribal E, Kazokoglu H (2003). The influence of sex hormones on ocular blood flow in women. Acta Ophthalmol Scand.

[37] Atalay E, Karaali K, Akar M, Ari ES, Simsek M, Atalay S (2005). Early impact of hormone replacement therapy on vascular hemodynamics detected via ocular colour Doppler analysis. Maturitas.

[38] Zhou X, Li F, Ge J, Sarkisian SR, Tomita H, Zaharia A (2007). Retinal ganglion cell protection by 17-beta-estradiol in a mouse model of inherited glaucoma. Dev Neurobiol.

[39] Russo R, Cavaliere F, Watanabe C, Nucci C, Bagetta G, Corasaniti MT (2008). 17Beta-estradiol prevents retinal ganglion cell loss induced by acute rise of intraocular pressure in rat. Prog Brain Res.

[40] Tsuzuki T, Okada H, Cho H, Shimoi K, Miyashiro H, Yasuda K (2013). Divergent regulation of angiopoietin-1, angiopoietin-2, and vascular endothelial growth factor by hypoxia and female sex steroids in human endometrial stromal cells. Eur J Obstet Gynecol Reprod Biol.

[41] Shen T, Gupta VK, Klistorner A, Chitranshi N, Graham SL, You Y (2019). Sex-specific effect of BDNF Val66Met genotypes on the progression of open-angle glaucoma. Invest Ophthalmol Vis Sci.

[42] Thurston G, Daly C (2012). The complex role of angiopoietin-2 in the angiopoietin-tie signaling pathway. Cold Spring Harb Perspect Med.

[43] Thomson BR, Heinen S, Jeansson M, Ghosh AK, Fatima A, Sung HK (2014). A lymphatic defect causes ocular hypertension and glaucoma in mice. J Clin Invest.

[44] Souma T, Tompson SW, Thomson BR, Siggs OM, Kizhatil K, Yamaguchi S (2016). Angiopoietin receptor TEK mutations underlie primary congenital glaucoma with variable expressivity. J Clin Invest.

[45] Morooka N, Futaki S, Sato-Nishiuchi R, Nishino M, Totani Y, Shimono C (2017). Polydom is an extracellular matrix protein involved in lymphatic vessel remodeling. Circ Res.

[46] Jackson AP, Eastwood H, Bell SM, Adu J, Toomes C, Carr IM (2002). Identification of microcephalin, a protein implicated in determining the size of the human brain. Am J Hum Genet.

